# Surgical outcomes and optimal approach to treatment of aortic valve endocarditis with aortic root abscess

**DOI:** 10.1111/jocs.16464

**Published:** 2022-04-05

**Authors:** William M. Harris, Shubhra Sinha, Massimo Caputo, Gianni D. Angelini, Eltayeb M. Ahmed, Cha Rajakaruna, Umberto Benedetto, Hunaid A. Vohra

**Affiliations:** ^1^ Bristol Heart Institute University Hospitals Bristol NHS Foundation Trust Bristol UK

**Keywords:** aortic root abscess, aortic root replacement, infective endocarditis, patch reconstruction

## Abstract

**Objectives:**

To evaluate the impact of aortic root abscess (ARA) on the postoperative outcomes of surgically managed infective endocarditis (IE) and to inform optimal surgical approach.

**Methods:**

Between 2009 and 2020, 143 consecutive patients who underwent surgical management for aortic‐valve IE were included in a retrospective cohort study. Multivariable and propensity‐weighted analyses were used to adjust for demographic imbalances between those without (*n* = 93; NARA) and with an ARA (*n* = 50). Additionally, empirical subgroup analysis appraised the two most used surgical techniques; patch reconstruction (PR) and aortic root replacement (ARR).

**Results:**

Demographic characteristics were similar between ARA and NARA except for logistic EuroSCORE, previous valve surgery, and multivalvular infection. In‐hospital mortality was 8% and 12% in NARA and ARA, respectively (*p* = .38), with mortality rates consistently nonsignificantly higher in ARA across all time periods. The overall reoperation rate was also higher in ARA (27% vs. 14%; *p* = .09) and ARA was shown to be associated with late reoperation (odds ratio [OR] = 2.74; 95% confidence interval [CI] = 1.18–6.36). Patients treated with an ARR showed a 16% increase in late mortality when compared with PR (40% vs. 24%; *p* = .27) and a 17% lower reoperation rate (14% vs. 31%; *p* = .24). Propensity‐weighted analysis identified ARR as a significant protective factor for reoperation (hazard ratio = 0.05; 95% CI = 0.01–0.34).

**Conclusions:**

The presence of an ARA in aortic valve endocarditis was not associated with significantly higher early and late mortality but is linked with a higher reoperation rate at our institution. ARR in ARA is protective from reoperation so should be considered best practice in this setting.

## 1 INTRODUCTION

Approximately half of all infective endocarditis (IE) patients are identified as high‐risk and undergo operative treatment.^1^ The presence of a paravalvular abscess is a crucial indication for surgical management^2^ due to its known association with in‐hospital mortality.^3^ However, the etiology, sequelae, and management of patients with paravalvular abscesses occurring in different regions of the heart are distinct.^4^ Aortic root abscesses (ARAs) are the most common type of paravalvular abscess,^5^, ^6^, ^7^ previously described as a catastrophic complication of IE. If left untreated, ARA can cause severe valvular dysfunction, heart block, pseudoaneurysm formation, and obstruction to coronary blood flow.^8^, ^9^ However, the impact of an ARA on early and late postoperative outcomes is not well defined due to the fragmented evidence base of retrospective cohort studies and the emergence of modern surgical techniques.^5^

**Figure 1:**
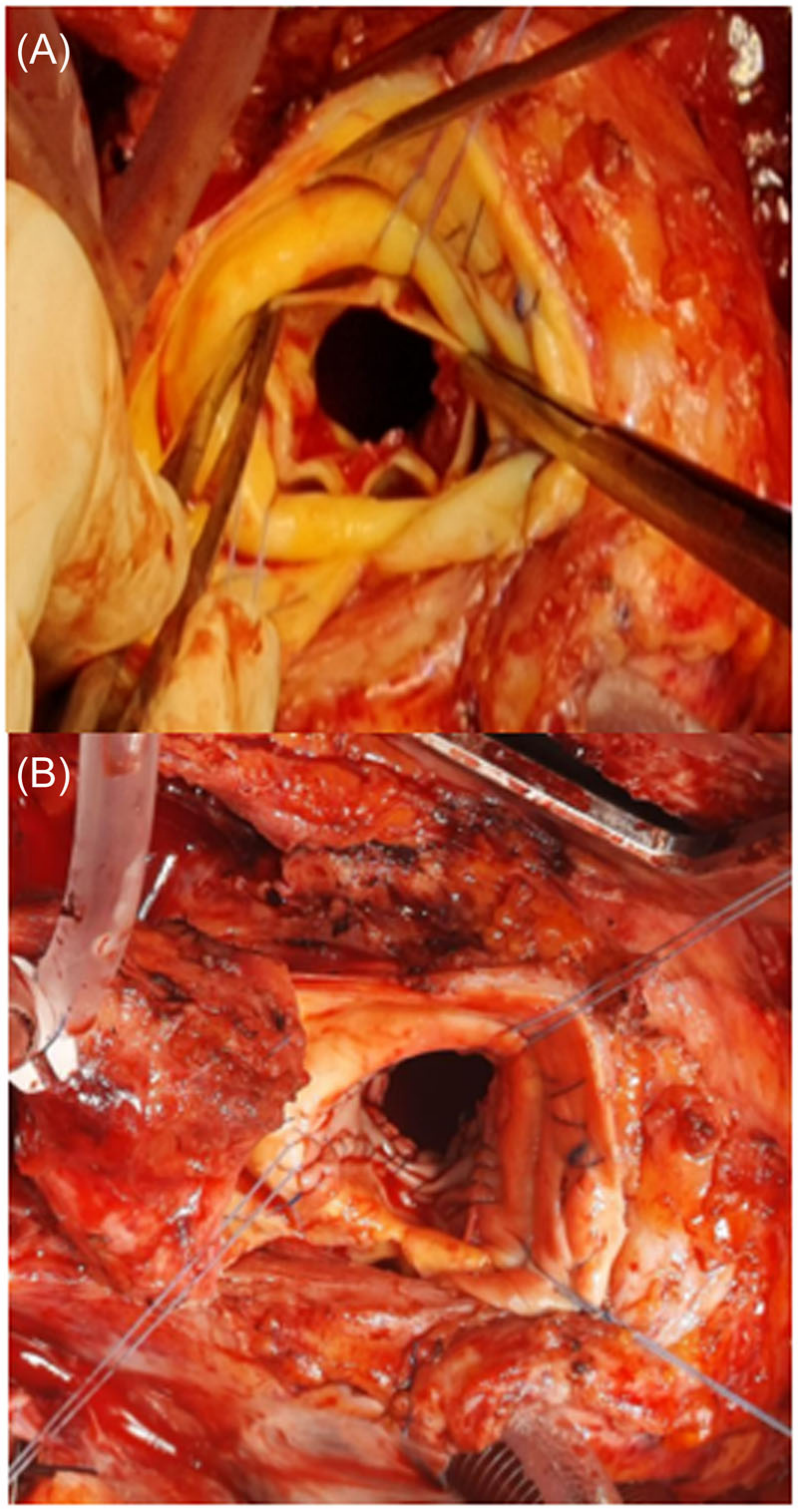
(A) Surgeon's view showing the ventricular surface of a bio‐prosthetic aortic valve studded with vegetations with florid endocarditis and root abscess. (B) Similar view after excision of the valve and repair of the abscess cavity with bovine pericardial patches in the noncoronary and left coronary sinuses.

The two primary goals for operative management of IE with or without an ARA are: (a) complete debridement of infective tissue and (b) reconstruction of cardiac morphology.^10^ The choice between these approaches currently relies on the extent of infection, surgeon or institutional preference, and demographic factors.^9^ In this study, we aim to establish the significance of ARA on postoperative outcomes and inform surgical decision‐making between the two most used techniques: patch reconstruction (PR; Figure 1) and aortic root replacement (ARR).

## 2 MATERIALS AND METHODS

Our empirical study was registered locally and was granted institutional ethical approval. As no patient identifiable information was collected, informed consent for participation was waived. Data was refined from a local surgical database at the Bristol Heart Institute, capturing the details of consecutive surgically managed patients with active IE of the aortic valve from 2009 to 2020. One hundred forty‐three patients who underwent first‐time surgical management for active IE were included in the study. These patients were divided into two groups according to the absence (*n* = 93; Group NARA) or presence (*n* = 50; Group ARA) of an ARA. Within Group ARA, 15 patients received an ARR, 29 were treated with PR, and in six cases, the aortic annulus was repaired using Prolene suture. Follow‐up data, including date of death, reoperation, and adverse events, were assimilated on review of a local surgical database and each patient's electronic health record (mortality and reoperation follow‐up 100% complete).

Categorical variables are expressed as percentages, with all raw data tabulated. Continuous variables are expressed as mean values with their associated standard deviations. Univariable comparisons for categorical variables were performed using the chi‐squared test and the independent Student's *t*‐test for continuous variables. The Fisher's exact test was preferred when dichotomous variables had an expected count of <5 in >20% of cells. Statistical significance was defined as a probability of *p* < .05, using two‐tailed *p* values. All preoperative variables found to be significantly different between the Groups NARA and ARA were identified as potential confounding factors hence were included in all subsequent multivariable analyses. Additionally, “a priori” selection was used to include any independent predictors of in‐hospital mortality identified in previous research. We performed statistical analysis on the outcomes of all‐cause in‐hospital mortality, late mortality, and late reoperation (defined as any additional surgical procedure performed in the follow‐up period). Multivariable logistic regression and inverse propensity treatment weighting (IPTW) analysis were used to calculate an adjusted OR for in‐hospital mortality, defined according to the Society of Thoracic Surgeons definition (including all causes of death occurring during the same hospitalization in which the surgery was performed, even after 30 days).^11^, ^12^ Cox proportional hazard modeling was used to calculate the adjusted hazard ratio (HR) for late mortality and late reoperation (measured from the date of surgery to follow‐up). To graphically represent differences in survival from death and reoperation, survival curves were constructed using the Kaplan–Meier method. The log‐rank test was used to assess differences in survival curves.

For our subgroup analysis, Group ARA was divided into patients managed with an ARR (*n* = 15) versus PR (*n* = 29). IPTW analysis adjusted for variables that were statistically different between the two treatment groups on univariable analysis. Additionally, Kaplan–Meier survival curves were constructed, demonstrating differences in mortality and reoperation over time. All statistical analyses were performed on SPSS 26 (IBM Corp).

## 3 RESULTS

### 3.1 Preoperative variables

On univariate comparison, there were no statistically significant differences in medical history, baseline characteristics, or comorbidities between the treatment groups. However, Group ARA had a significantly lower proportion of patients with previous aortic valve replacement (AVR; 16% vs. 33%; *p *≤ .01) and a higher rate of multivalvular infection (23% vs. 6%; *p* = .01). Additionally, the logistic EuroSCORE, was significantly higher in Group ARA (25.5 vs. 18.1; *p* = .04). Streptococcal infection was the most common infective agent (35%) but there were no significant differences in cultured microorganisms between Groups ARA and NARA (Table 1).

**Table 1:** Preoperative characteristics with univariate analysis

Preoperative variable	Total (*n* = 143)	No ARA (*n* = 93)	ARA (*n* = 50)	*p*
**Age (years)**	58.5 (SD ± 15.0)	58.3 (SD ± 13.6)	58.9 (SD ± 17.3)	.838
	Median – 60.6			
**Male**	78% (111/143)	77% (72/93)	78% (39/50)	.937
**BMI**	26.0 (SD ± 5.6)	26.0 (SD ± 5.3)	26.0 (SD ± 6.2)	.982
**Smoking**				.254
Never smoked	46% (63/137)	50% (45/90)	38% (18/47)	
Ex‐smoker	30% (41/137)	30% (27/90)	30% (14/47)	
Current smoker	24% (33/137)	20% (18/90)	32% (15/47)	
**Hypertension**	33% (46/141)	35% (32/92)	29% (14/49)	.454
**Neurological history**				.108
No history	84% (116/138)	82% (74/90)	88% (42/48)	
**Previous MI**	4% (6/139)	4% (4/91)	4% (4/48)	1.000
**Rhythm**				.166
Sinus rhythm	88% (124/141)	91% (84/92)	82% (40/49)	
Atrial fibrillation/flutter	3% (4/141)	3% (3/92)	2% (1/49)	
Complete heart block/pacing	6% (9/141)	4% (3/92)	10% (5/49)	
**Cardiac surgical history**				.410
No previous surgery	87% (120/138)	90% (81/90)	81% (39/48)	
**Diabetes**	6% (9/143)	5% (5/93)	8% (4/50)	.720
**Creatinine**	120.4 (SD ± 69.1)	115 (SD ± 56.6)	130 (SD ± 87.4)	.274
**IVDU**	13% (12/92)	11% (7/63)	17% (5/29)	.417
**LVEF**				.865
Poor (<30%)	9% (13/143)	9% (8/93)	10% (5/50)	
**Organism grown**				.227
Culture negative	32% (45/139)	34% (32/93)	26% (13/50)	.229
*Streptococci*	35% (49/139)	34% (30/89)	38% (19/50)	.611
*Staphylococci*	18% (25/139)	18% (16/89)	18% (9/50)	.997
*Enterococci*	6% (8/139)	8% (7/89)	2% (1/50)	.154
*Escherichia coli*	2% (3/139)	1% (1/89)	4% (2/50)	.263
**Operative urgency**				.115
Urgent	58% (83/143)	58% (54/92)	58% (29/50)	
Emergency	23% (33/143)	18% (17/93)	32% (16/50)	
**Previous AVR**	19% (26/137)	11% (10/89)	33% (16/48)	**.002**
**Aortic explant**				**.004**
Native	81% (111/137)	89% (79/89)	67% (32/48)	
Mechanical	7% (10/137)	6% (5/89)	10% (5/48)	
Biological	12% (16/137)	6% (5/89)	23% (11/48)	
**Logistic EuroSCORE**	20.7 (SD ± 18.9)	18.1 (SD ± 16.6)	25.5 (SD ± 22.0)	**.040**
**Multivalvular infection**	17% (24/143)	23% (21/93)	6% (3/50)	**.011**

*Note*: Bold indicates statistical significance. Continuous variables are given as mean values (with associated standard deviation) unless stated otherwise. Categorical variables are given as percentages (with associated raw data).

Abbreviations: ARA, aortic root abscess; AVR, aortic valve replacement; BMI, body mass index; IVDU, intravenous drug user; LVEF, left ventricular ejection fraction; MI, myocardial infarction; SD, standard deviation.

### 3.2 Operative variables

As the surgical approach is inherently different in patients with and without an ARA, there were several discrepancies in operative data. This included statistically significant differences in the type of aortic valve implant used (*p* = .02) and whether an aortic root operation was performed (*p *≤.01; Table 2). Homograft and biological valve implants were preferred in Group ARA as they are known to have lower re‐infection rates compared with mechanical valves in cases of fulminant infection.^13^ Additionally, a longer cumulative cross‐clamp time (118.7 ± 59.1 vs. 94.6 ± 42.9 min) and cumulative bypass time (168.1 ± 87.9 vs. 132.5 ± 64.9 min) in Group ARA reflects the increased complexity of ARA surgery.

**Table 2:** Operative characteristics with univariate analysis

Operative variable	Total (*n* = 143)	No ARA (*n* = 93)	ARA (*n* = 50)	*p*
**Aortic valve implant type**				**.019**
Mechanical	28% (39/139)	29% (26/89)	26% (13/50)	
Biological	67% (93/139)	70% (62/89)	62% (31/50)	
Homograft	5% (7/139)	1% (1/89)	12% (6/50)	
**Aortic valve ring size**	22.8 (SD ± 2.5)	22.8 (SD ± 2.6)	22.8 (SD ± 2.5)	.796
**Aortic root operation**				**<.001**
No root operation	55% (79/143)	85% (79/93)	0% (0/50)	
6.0 Prolene sutures	4% (6/143)	0% (0/93)	12% (6/50)	
PR	23% (33/143)	3% (4/93)	54% (29/50)	
ARR	15% (22/143)	5% (7/93)	30% (15/50)	
**Cumulative bypass time (min)**	144.5 (SD ± 75.0)	132.5 (SD ± 64.9)	168.1 (SD ± 87.9)	**.011**
**Cumulative cross clamp (min)**	102.7 (SD ± 50.0)	94.6 (SD ± 42.9)	118.7 (SD ± 59.1)	**.009**

*Note*: Bold indicates statistical significance. Continuous variables are given as mean values (with associated standard deviation) unless stated otherwise. Categorical variables are given as percentages (with associated raw data).

Abbreviations: ARA, aortic root abscess; ARR, aortic root replacement; PR, patch reconstruction.

### 3.3 Postoperative outcomes

The presence of an ARA was associated with several differences in postoperative outcomes. Importantly, mortality rates were consistently higher in Group ARA across all time periods, including in‐hospital (12% vs. 8%; *p* = .38), 1‐year (20% vs. 11%; *p* = .13), and late mortality (30% vs. 19%; *p* = .15; Table 3). Additionally, the reoperation rate was markedly higher in Group ARA (27% vs. 14% *p* = .085), driven by a high incidence of structural deterioration in this group (18% vs. 3%).

**Table 3:** Postoperative outcomes with univariate analysis

Postoperative variable	Total (*n* = 143)	No ARA (*n* = 93)	ARA (*n* = 50)	*p*
**Stroke**	7% (7/101)	8% (5/65)	6% (2/36)	.686
**Dialysis**	9% (9/102)	5% (3/65)	16% (6/37)	.069
**Deep sternal wound infection**	2% (2/85)	0% (0/57)	8% (2/28)	.106
**Late reoperation**	19% (26/139)	14% (13/90)	27% (13/49)	.081
**Reoperation**				**.035**
No reoperation	81% (112/139)	84% (76/90)	73% (36/49)	
Reoperation for structural deterioration	9% (12/139)	3% (3/90)	18% (9/49)	
**In‐hospital mortality**	9% (13/143)	8% (7/93)	12% (6/50)	.375
**Late mortality**	23% (33/143)	19% (18/93)	30% (15/50)	.150
**Follow‐up (months)**	Range: 0–142	49.6 (SD ± 41.1)	47.0 (SD ± 40.1)	.717
	Median: 43.0			

*Note*: Bold indicates statistical significance. Continuous variables are given as mean values (with associated standard deviation) unless stated otherwise. Categorical variables are given as percentages (with associated raw data).

Abbreviation: ARA, aortic root abscess.

#### 3.3.1 Aortic root abscess versus no aortic root abscess

Unadjusted data suggested an inconclusive trend toward increased risk of in‐hospital mortality given the presence of an ARA (odds ratio [OR] = 1.68; 95% confidence interval [CI] = 0.53–5.29). Additionally, our multivariable logistic regression model gave no further insight into this relationship (Table 4). However, it was notable that on propensity‐weighted statistical adjustment, there was a subtle shift toward the null value (OR = 1.29; 95% CI = 0.38–4.37).

**Table 4:** Unadjusted OR figures for in‐hospital mortality alongside Logistic regression and propensity‐weighted analysis

	Unadjusted OR		Logistic regression		Propensity‐weighted regression	
	OR (95% CI)	*p*	OR (95% CI)	*p*	OR (95% CI)	*p*
ARA	1.68 (0.53–5.29)	.379	2.07 (0.42–10.18)	.371	1.29 (0.38–4.37)	.677
Logistic EuroSCORE	1.05 (1.02–1.08)	**<.001**	1.05 (1.02–1.09)	**.001**		
Previous AVR	1.38 (0.35–5.42)	.642	0.64 (0.10–3.99)	.629		
Surgery pre‐2016	2.56 (0.79–8.26)	.116	2.14 (0.53–8.56)	.284		
Multivalvular Infection	5.33 (1.61–17.68)	**.006**	12.98 (2.55–65.98)	**.002**		
Staphylococcal infection	1.47 (0.38–5.79)	.579	0.82 (0.14–5.02)	.834		
NYHA Class ≥ 3	1.12 (0.55–2.28)	.764	0.72 (0.16–3.13)	.658		

*Note*: Bold indicates statistical significance.

Abbreviations: ARA, aortic root abscess; AVR, aortic valve replacement; CI, confidence interval; NYHA, New York Heart Association; OR, odds ratio.

**Figure 2:**
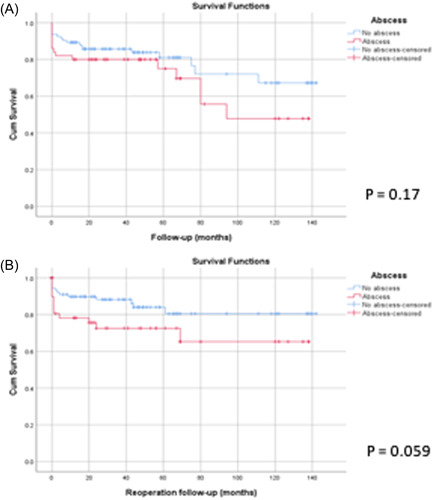
Kaplan–Meier curves show (A) survival from death and (B) survival from reoperation in patients with and without an ARA. ARA, aortic root abscess.

Although our Kaplan–Meier curve demonstrated a consistently higher mortality rate in Group ARA, this association was not statistically significant on cox regression statistical modeling (OR = 1.67; 95% CI = 0.77–3.53; *p* = .19) or on review of the log‐rank test (*p* = .17; Figure 2A). Nevertheless, we were able to identify logistic EuroSCORE (OR = 1.05; 95% CI = 1.02–1.08; *p *≤ .01) and multivalvular infection (OR = 5.33; 95% CI = 1.61–17.68; *p* = .02) as risk factors for both in‐hospital and late mortality (Table 5). Additionally, the presence of an ARA was shown to be strongly associated with late reoperation (OR = 2.74; 95% CI = 1.18–6.36; *p* = .02), the only identifiable independent reoperation in our empirical analysis (Table 5). Further, though the log‐rank test did not reach clear‐cut statistical significance (*p* = .06), Figure 2B depicts the strong association between ARA and high reoperation rates.

**Table 5:** Cox proportional hazard analysis for late mortality and reoperation

	Late mortality		Late reoperation	
	RR (95% CI)	*p*	RR (95% CI)	*p*
ARA	1.65 (0.77–3.53)	.185	2.74 (1.18–6.36)	**.019**
logistic EuroSCORE	1.04 (1.02–1.05)	**<.001**	1.02 (<1.00–1.04)	.096
Previous AVR	0.43 (0.16–1.19)	.105	0.37 (0.10–1.33)	.128
Multivalvular Infection	2.80 (1.19–6.58)	**.018**	1.96 (0.70–5.52)	.203
Surgery pre‐2016	1.82 (0.79–4.18)	.158	0.58 (0.24–1.38)	.218

*Note*: Bold indicates statistical significance.

Abbreviations: ARA, aortic root abscess; AVR, aortic valve replacement; CI, confidence interval; RR, risk ratio.

#### 3.3.2 Patch reconstruction versus aortic root replacement

**Figure 3:**
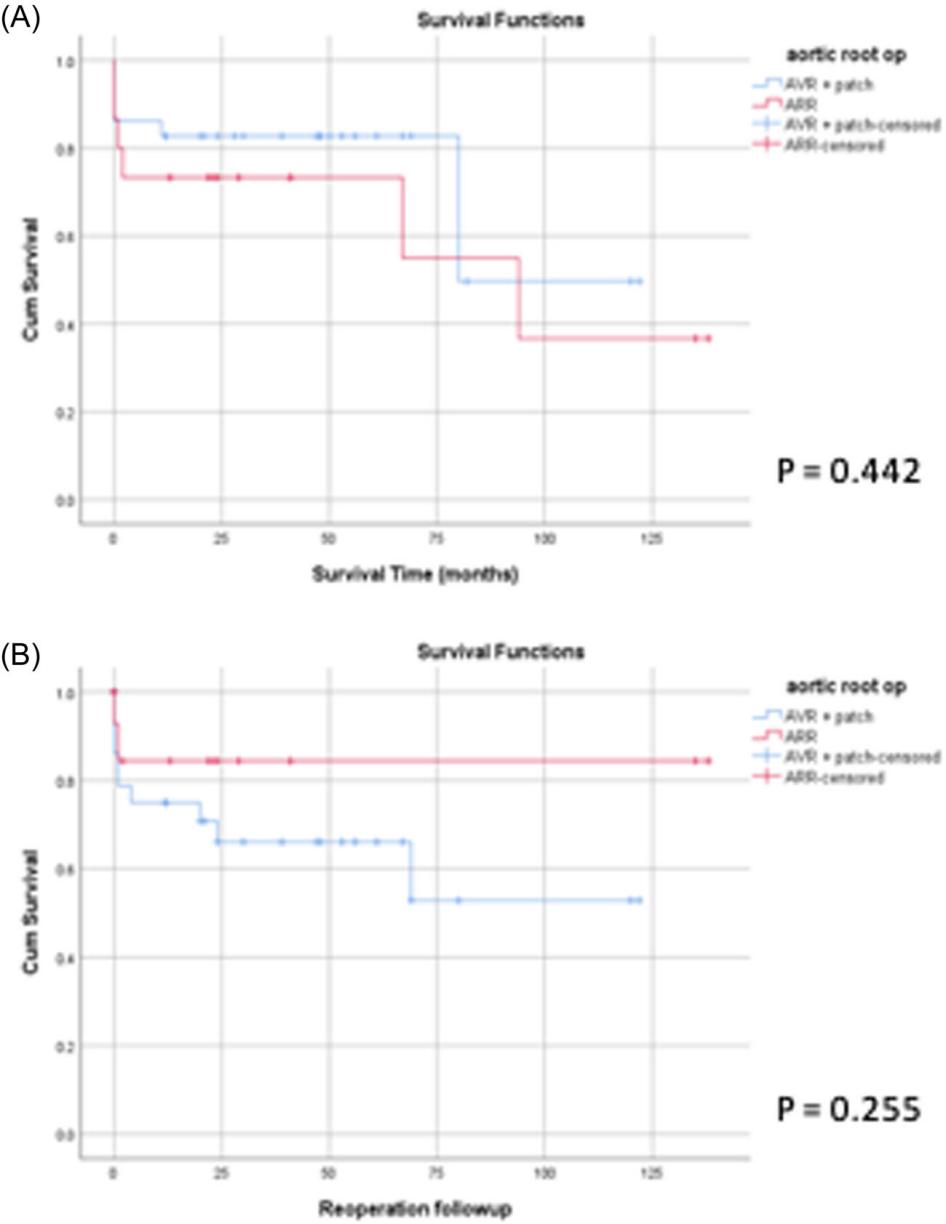
Kaplan–Meier curves show (A) survival from death and (B) survival from reoperation for ARA patients treated with an ARR versus PR. ARA, aortic root abscess; PR, patch reconstruction.

Preoperative data with univariable analysis were tabulated (Table S1). In our subgroup analysis, we found that Group PR was composed of a greater proportion of males (89% vs. 60%; *p* = .03) while group ARR had a higher incidence of previous AVR (64% vs. 24%; *p* = .006) and staphylococcal infection (40% vs. 10%; *p* = .04; Table S1). There were also several notable differences in the unadjusted postoperative outcomes for patients treated with PR versus ARR (Table S3). Patients treated with an ARR showed a 16% increase in late mortality when compared with PR (40% vs. 24%; *p* = .27) and a 17% lower reoperation rate (14% vs. 31%; *p* = .24) (Table S2). Figure 3 demonstrates that ARRs have a stronger association with lower reoperation rates (*p* = .255) than higher mortality (*p* = .442). However, both associations failed to reach statistical significance with a moderate likelihood that these relationships are due to random chance. Nevertheless, on propensity‐weighted analysis, ARR was identified as a significant protective factor for reoperation (HR = 0.05; 95% CI = 0.01–0.34) while the same statistical model yielded a poorly defined relationship between surgical technique and late mortality (HR = 2.60; 95% CI = 0.39–17.29; Table S3).

## 4 DISCUSSION

In our institution, we found that ARA was not associated with a clear‐cut increased risk of in‐hospital mortality or late mortality. This may suggest that modern standards of care have improved the survival rates for endocarditis complicated by ARA. However, ARA remains to be a large contributor to the surgical workload (responsible for one third of aortic valve endocarditis surgeries).^14^, ^15^, ^16^, ^17^

A salient finding from our postoperative analysis was a 13% higher reoperation rate in patients with an ARA (Table 3). Evidence of this relationship was strengthened by our Cox proportional hazard analysis, which identified ARA as an independent risk factor for reoperation (HR = 2.74; 95% CI = 1.18–6.36; *p* = .12; Table 5). However, this finding was not consistent with other centers.^15^, ^17^ Differences in institutional preference for ARR versus PR may explain this finding as preceding reports have suggested that ARR accomplishes more comprehensive removal of infected tissue and reconstruction of cardiac morphology compared with PR and so can achieve lower rates of reinfection and graft deterioration.^18^, ^19^ Therefore, our high reoperation rate may reflect our institutional practice of performing ARR in one third of ARA cases.

Subgroup analysis of group ARR versus group PR provides further insight into this association. Patients who received ARR were associated with a 17% lower risk of late reoperation compared with PR, and our propensity‐weighted analysis provided additional evidence to suggest that ARR is associated with a lower reoperation rate compared with PR (RR = 0.05; 95% CI = 0.01–0.34). Our results coincide with the Chen et al.^8^ meta‐analysis which also showed that patients treated with an ARR were at a lower risk of reoperation at 1‐year follow‐up. Therefore, there is growing evidence to suggest that ARR is associated with advantageous reoperation rates, a hypothesis that is also intuitive and backed by well‐established surgical principles.

Our analysis did not show a significant association between ARR and postoperative mortality. This is in agreement with the aforementioned meta‐analysis,^8^ which reported that, although positively correlated, ARR was not a significant risk factor for increased early mortality (OR = 1.30; 95% CI = 0.84–2.00; *p* = .66).^8^ In our institution, group ARR was not associated with an increased risk of mortality even despite a higher logistic EuroSCORE, a finding similar to the report by Leontyev et al.^20^ Therefore, evidence of subtle trends of ARR‐associated mortality in observational studies is likely to be confounded by the higher risk population on which they were performed.^20^, ^21^ This consideration does not explain lower reoperation rates observed in patients treated by ARR, so the disparity in reoperation rates can still be attributed to the operation itself. Therefore, we may be able to recommend a lower threshold for ARR in cases with less extensive damage to the aortic root to reduce reoperation rates for ARA patients safely. This change in practice could constitute a step forward in precise surgical decision‐making for IE patients, bringing the management of this disease into the realm of evidence‐based medicine. Nevertheless, more research is required in this area to establish the equity of ARR and PR in the treatment of ARA.

Our single‐center experience has highlighted the limitations inherent to retrospective observational studies. The sensitivity of logistic regression and Cox proportional hazard models are weakened when used on cohorts of patients with a low frequency of expected events and a high number of covariates relative to sample size. This could be overcome with the emerging use of merged databases. Additionally, we used logistic EuroSCORE, which has now been replaced by EuroSCORE II.

## 5 CONCLUSION

The causal relationship between ARA and mortality remains uncertain and may be more subtle than previously thought due to the influence of confounding factors, which were not adequately addressed by preceding reports. We have also shown that the ARR technique is associated with an advantageous postoperative profile compared with PR. Clearly, more research is needed in this area with larger numbers but based on our findings and other published literature; ARR could be recommended as the best practice treatment for IE complicated by ARA.

## CONFLICTS OF INTEREST

The authors declare no conflicts of interest.

## ETHICS STATEMENT

Our study was granted institutional approval and did not require International Review Board.

## AUTHORS CONTRIBUTIONS

Hunaid A. Vohra and William M. Harris conceived the project idea and designed the analysis. William M. Harris collected the data with the help of Shubhra Sinha. William M. Harris performed the analysis under the supervision of Shubhra Sinha and Hunaid A. Vohra. William M. Harris wrote the manuscript with input from Shubhra Sinha, Massimo Caputo, Gianni D. Angelini, Eltayeb M. Ahmed, Cha Rajakaruna, Umberto Benedetto, and Hunaid A. Vohra. All authors reviewed the results and approved the final version of the manuscript.

## Supporting information

Supplementary information.
