# Supplementary material for: Surgical outcomes and optimal approach to treatment of aortic valve endocarditis with aortic root abscess
Source: J Card Surg. 2022 Apr 5;37(7):1917–25. doi: 10.1111/jocs.16464 (PMC9321057; doi:10.1111/jocs.16464)
Supplement: Supplementary file 1 — Supplementary information. [file JOCS-37-1917-s001.docx]

**Supplementary Material**

**Supplementary Table 1 -** Preoperative outcomes and univariate analysis PR vs ARR

| Demographic Characteristic | PR  (n = 29) | ARR  (n=15) | P value |  |
| --- | --- | --- | --- | --- |
|  |  |  |  |  |
| **Age (years)** | 56.9 (SD +- 17.2) | 60.5 (SD +- 19.1) | 0.542 |  |
| **Male** | 89% (24/27) | 60% (9/15) | **0.029** |  |
| **BMI** | 26.1 (SD +- 5.6) | 27.2 (SD +- 7.9) | 0.619 |  |
| **Smoking** |  |  |  |  |
| Never smoked | 41% (11/27) | 27% (4/15) | 0.641 |  |
| **Hypertension*** | 22% (6/27) | 40% (6/15) | 0.222 |  |
| **History of CVA** | 15% (4/27) | 13% (2/15) | 1 |  |
| **Previous MI** | 0% (0/28) | 14% (2/14) | 0.106 |  |
| **Abnormal heart rhythm** | 26% (6/29) | 21% (3/14) | 1 |  |
| **Creatinine** | 118.5 (SD +- 61.8) | 140.8 (SD +- 128.8) | 0.535 |  |
| **IVDU** | 14% (4/29) | 7% (1/15) | 0.647 |  |
| **LVEF** |  |  |  |  |
| Poor (LVEF < 30%) | 7% (2/29) | 13% (2/15) | 0.315 |  |
| **Organism grown** |  |  |  |  |
| Culture negative | 28% (8/29) | 13% (2/15) | 0.452 |  |
| Streptococcal | 45% (14/29) | 20% (3/15) | 0.185 |  |
| Staphylococcal | 10% (3/29) | 40% (6/15) | **0.044** |  |
| **Operative urgency** |  |  |  |  |
| Urgent | 48% (14/29) | 67% (10/15) | 0.072 |  |
| Emergency | 44% (13/29) | 13% (2/15) |  |  |
| **Previous AVR** | 21% (6/28) | 64% (9/14) | **0.006** |  |
| **Logistic EuroSCORE** | 21.9% (SD +- 19.9) | 36.3% (SD +- 25.4) | 0.068 |  |

Key - Bold indicates statistical significance. For continuous variables, values given as mean value (standard deviation). For categorical variable, values given as % (raw data). SD, Standard Deviation; PR, Patch Reconstruction; ARR, Aortic Root Replacement; BMI, Transient Ischaemic Attack; CVA, cerebrovascular Accident; MI, Myocardial Infarction; IVDU, Intravenous Drug User; LVEF, Left Ventricular Ejection Fraction; AVR, Aortic Valve Replacement

**Supplementary Table 2 -** Postoperative outcomes and univariate analysis PR vs ARR

| Postoperative outcome | PR (n = 29) | ARR (n=15) | P value |
| --- | --- | --- | --- |
| **Stroke** | 0% (0/19) | 8% (1/12) | 0.210 |
| **Dialysis** | 20% (3/15) | 21% (3/14) | 0.732 |
| **Wound infection** | 11% (2/19) | 0% (0/8) | 0.340 |
| **Late reoperation** | 31% (9/29) | 14% (2/14) | 0.238 |
| **In-hospital mortality** | 10% (3/29) | 20% (3/15) | 0.376 |
| **Late mortality** | 24% (7/29) | 40% (6/15) | 0.274 |
| **Follow up (months)** | 43.9 (33) | 42 (46.4) | 0.874 |

Key - Bold indicates statistical significance. For continuous variables, values given as mean value (standard deviation). For categorical variable, values given as % (raw data). PR, Patch Reconstruction; ARR, Aortic Root Replacement

**Supplementary Table 3**: IPTW analysis of late mortality and reoperation

|  | Late Mortality | | Late Reoperation | |
| --- | --- | --- | --- | --- |
|  | RR (95% C.I.) | P value | RR (95% C.I.) | P value |
| ARR | 2.60 (0.39-17.29) | 0.322 | 0.05 (0.01-0.34) | **0.002** |

Key – RR, Risk Ratio; CI, Confidence Interval; ARR, Aortic Root Replacement
